# Sequencing and characterization of Varicella-Zoster virus vaccine strain SuduVax

**DOI:** 10.1186/1743-422X-8-547

**Published:** 2011-12-16

**Authors:** Jong Ik Kim, Gyoo Seung Jung, Yu Young Kim, Ga Young Ji, Hyung Seok Kim, Wen Dan Wang, Ho Sun Park, Song Yong Park, Geun Hee Kim, Shi Nae Kwon, Keon Myung Lee, Jin Hyun Ahn, Yeup Yoon, Chan Hee Lee

**Affiliations:** 1 Department of Microbiology, Chungbuk National University, Cheongju, South Korea; 2 Department of Micorbiology, College of Medicine, Yeungnam University, Daegu, South Korea; 3 Mogam Biotechnology Research Institute, Yongin, South Korea; 4 Green Cross Company, Yongin, South Korea; 5 Department of Molecular Cell Biology, Sungkyunkwan University School of Medicine, Suwon, South Korea; 6 Department of Computer Science, Chungbuk National University, Cheongju, South Korea

**Keywords:** Varicella-zoster virus, SuduVax, Genome, Phylogeny

## Abstract

**Background:**

Varicella-zoster virus (VZV) causes chickenpox in children and shingles in older people. Currently, live attenuated vaccines based on the Oka strain are available worldwide. In Korea, an attenuated VZV vaccine has been developed from a Korean isolate and has been commercially available since 1994. Despite this long history of use, the mechanism for the attenuation of the vaccine strain is still elusive. We attempted to understand the molecular basis of attenuation mechanism by full genome sequencing and comparative genomic analyses of the Korean vaccine strain SuduVax.

**Results:**

SuduVax was found to contain a genome that was 124,759 bp and possessed 74 open reading frames (ORFs). SuduVax was genetically most close to Oka strains and these Korean-Japanese strains formed a strong clade in phylogenetic trees. SuduVax, similar to the Oka vaccine strains, underwent T- > C substitution at the stop codon of ORF0, resulting in a read-through mutation to code for an extended form of ORF0 protein. SuduVax also shared certain deletion and insertion mutations in ORFs 17, 29, 56 and 60 with Oka vaccine strains and some clinical strains.

**Conclusions:**

The Korean VZV vaccine strain SuduVax is genetically similar to the Oka vaccine strains. Further comparative genomic and bioinformatics analyses will help to elucidate the molecular basis of the attenuation of the VZV vaccine strains.

## Background

Varicella-zoster virus (VZV) is an alpha-herpesvirus and the cause of chickenpox (varicella) and shingles (zoster). Chickenpox is characterized by fever and generalized rash, and is most prevalent in children due to primary infection. VZV can establish a latent infection in nerve cells of dorsal root ganglia and its reactivation from latency causes shingles in older adults and in immunocompromised people.

Isolation and propagation of VZV in cell culture was first reported in 1953 [1], and the first determination of the complete nucleotide sequence was made from the Dumas strain [2]. As of August 2010, complete nucleotide sequences had been determined and were available from NCBI GenBank database from 23 VZV strains including three vaccine strains derived from the Oka strain. Comparison of the full nucleotide sequences of clinical with vaccine strains has enabled researchers to suggest putative regions that might be responsible for attenuation in vaccine strains [3-6].

In Korea, the pharmaceutical company GCC has been manufacturing an attenuated VZV vaccine for chickenpox since 1994. The live-attenuated vaccine strain, SuduVax^®^, was obtained through serial passage of wild-type virus in cell culture. The original wild-type virus was isolated in primary human embryonic lung (HEL) cell culture from a 33-month-old boy with chickenpox in 1989 in Seoul, Korea [7]. The virus was attenuated by 10 passages in HEL cells, 12 passages in guinea pig embryonic lung cells, and passaged five times in HEL cells to prepare an attenuated strain, designated MAV06, for vaccine production [8]. The attenuated viruses were stored in liquid nitrogen (master virus banks). Working virus banks are routinely produced after five passages of master virus bank stocks in HEL cells. The final vaccine (SuduVax) is manufactured after passaging of the working virus bank five times in HEL cells.

SuduVax has been marketed in Korea since 1994 and internationally since 1998. Although the efficacy and safety of SuduVax have been proved in the marketplace, molecular studies explaining the mechanism of attenuation or the efficacy of the vaccine have not been available. In this study, the complete nucleotide sequence of SuduVax was determined and compared with those of 23 VZV strains whose full genomic sequences are registered in the NCBI GenBank database.

## Results

### Overall genome structure of the Korean vaccine strain SuduVax

**Figure 1:**
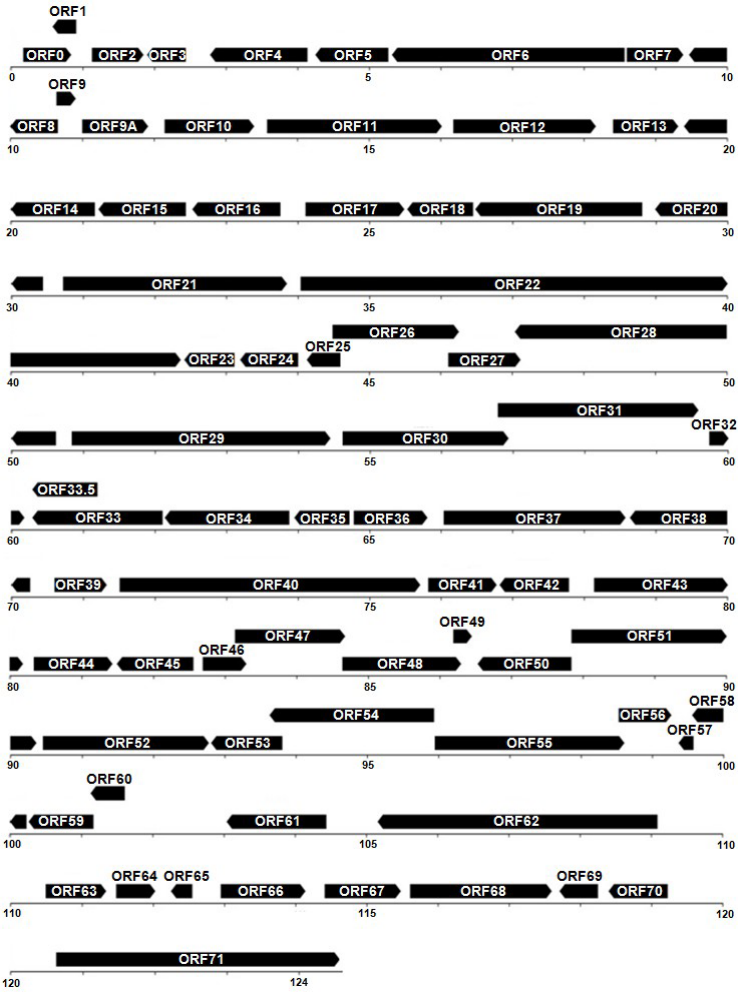
**ORF map of the VZV strain SuduVax**. The direction of the arrows indicates the direction of transcription.

The genome of the VZV strain SuduVax was determined to be 124,759 bp. The architecture of the SuduVax genome is typical of VZV in that the genome could be divided into TRL, UL, IRL, IRS, US and TRS (88, 104,799, 88, 7,276, 5,232, and 7276 bp, respectively). The G + C content of the SuduVax genome is approximately 46.1%. The lengths of the genome, lengths of each region and the G + C contents are very similar among the 24 VZV strains analyzed in this study (Table 1). The SuduVax genome contains 74 ORFs. Of these 64 are UL genes and four are US genes. Three genes in IRS (ORFs 62-64) are inversely repeated in TRS (ORFs 69-71). Of the 74 ORFs, 39 are in the forward direction and 35 are in the reverse direction. The directions of ORFs are 100% conserved among the analyzed VZV strains. The ORF map of strain SuduVax is presented in Figure 1.

**Table 1:** Information of the VZV strains analyzed in this study

Strain	Accession Number	Country	Length (bp)							%G+C
				
			Genome	TRl	Ul	IRl	IRs	Us	TRs	
Dumas	NC001348	Netherlands	124,884	88	104,836	88	7,320	5,232	7,320	46.0
M2DR	DQ452050	Morocco	124,770	89	104,719	89	7,320	5,232	7,321	46.0
CA123	DQ457052	USA	124,771	100	104,698	98	7,322	5,232	7,321	46.0
SD	DQ479953	USA	125,087	88	104,787	88	7,446	5,232	7,446	46.1
Kel	DQ479954	USA	125,374	88	104,857	88	7,555	5,232	7,554	46.2
11	DQ479955	Canada	125,370	88	104,906	88	7,529	5,232	7,527	46.2
22	DQ479956	Canada	124,868	88	104,689	88	7,386	5,232	7,385	46.0
03-500	DQ479957	Canada	125,239	88	105,299	88	7,266	5,232	7,266	46.1
36	DQ479958	Canada	125,030	88	104,850	88	7,387	5,232	7,385	46.1
49	DQ479959	Canada	125,041	88	104,916	88	7,358	5,232	7,359	46.1
8	DQ479960	Canada	125,451	89	105,020	88	7,510	5,232	7,512	46.2
32p5	DQ479961	USA	124,945	88	104,760	88	7,389	5,232	7,388	46.1
32p22	DQ479962	USA	125,084	88	104,791	88	7,443	5,232	7,442	46.1
32p72	DQ479963	USA	125,169	88	104,870	88	7,446	5,232	7,445	46.1
NH29_3	DQ674250	USA	124,811	87	104,766	87	7,320	5,230	7,321	46.0
SVETA	EU154348	Russia	124,813	87	104,772	87	7,319	5,230	7,318	46.0
MSP	AY548170	USA	124,883	88	104,848	88	7,313	5,232	7,314	46.0
BC	AY548171	Canada	125,459	88	105,326	88	7,363	5,231	7,363	46.2
HJ0	AJ871403	Germany	124,928	89	104,752	89	7,335	5,230	7,433	46.0
pOka	AB097933	Japan	125,125	88	104,798	88	7,463	5,225	7,463	46.1
vOka	AB097932	Japan	125,078	88	104,822	88	7,427	5,232	7,421	46.1
VarilRix	DQ008354	Japan	124,821	88	104,761	88	7,326	5,231	7,327	46.1
VariVax	DQ008355	Japan	124,815	88	104,758	88	7,324	5,232	7,325	46.1
SuduVax	This study	Korea	124,759	88	104,799	88	7,276	5,232	7,276	46.1

### Phylogenetic analysis

**Figure 2:**
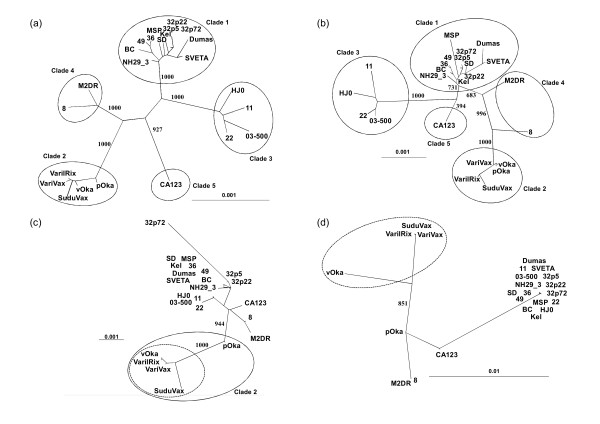
**Phylogenetic analysis of 24 VZV strains**. Nucleotide or amino acid sequences were multiple-aligned using ClustalW program (ver 2.0.1) and the resulting *.phy files were used to construct phylogenetic trees using maximum-likelihood (ML) or neighbor-joining (NJ) methods in Phylip package (version 3.69). (**a**) ML tree based on full nucleotide sequences. (**b**) ML tree based on non-coding sequences. (**c**) NJ tree based on the nucleotide sequences of ORF62, showing clear separation of vaccine strains from pOka within clade 2. (**d**) NJ tree based on the nucleotide sequences of ORF1. Vaccine strains are separated from clinical strains, but formation of clade 2 is not evident.

Phylogenetic trees were constructed using the full nucleotide sequences of SuduVax and 23 VZV strains whose full genomic DNA sequences are known. As shown in an unrooted tree generated by maximum-likelihood method, SuduVax and four Oka strains (pOka, vOka, VarilRix, VariVax) formed a clade and strains M2DR and 8 formed an adjacent clade (Figure 2a). These two clades were joined with the clade whose member was the strain CA123 only. Strains 11, 22, 03-500 and HJ0 formed another clade and the rest of the clinical strains formed the last clade. Almost identical topology was observed in a tree generated by neighbour-joining method (data not shown) and Bayesian method [9]. SuduVax together with Oka strains formed a distinctive clade, corresponding to clade 2 proposed by the VZV Nomenclature Meeting 2008 [10]. When trees were constructed with concatenated coding nucleotide sequences (ORF) or amino acid sequences, similar tree topologies were obtained (data not shown). Next, we tried to build phylogenetic trees using non-coding sequences. Again, SuduVax grouped with four Oka strains, forming clade 2 (Figure 2b). One notable difference between the trees built by full or coding sequences and the tree built by non-coding sequences was the location of pOka, the parental Oka strain from which vaccine strain vOka was derived. While pOka was located between the four vaccine strains and 19 clinical strains in the tree built by full or coding sequences, pOka was buried among the vaccine strains in tree built by non-coding sequences (compare Figures 2a, b). In other words, four vaccine strains (vOka, VarilRix, VariVax, and SuduVax) formed a subclade within the clade 2 in the trees built by full or coding sequences (bootstrap value = 1,000 in neighbour-joining trees), but not in the tree built by non-coding sequences.

In order to find which ORFs are important in distinguishing vaccine strains from clinical strains, further phylogentic analyses using individual ORF were performed. Of the 74 phylogenetic trees, 12 ORF trees exhibited clear branches leading to a formation of clusters consisting of vaccine strains. These 12 ORFs included ORF 0, 1, 6, 18, 31, 35, 39, 59, 62, 64, 69 and 71 (Figure 2c). The bootstrap values for vaccine clusters were greater than 640. In majority of ORF trees, vaccine clusters formed subclades within clade 2. However, in phylogenetic trees based on ORFs 1, 18, 39 and 59, branches leading to clade 2 were not present or very short with low bootstrap values (Figure 2d). Thus, the vaccine strains did not always form a subclade within clade 2.

Evolutionary relationships between the Korean vaccine strain SuduVax and other VZV strains were investigated by calculating genetic distances among the 24 VZV strains. As a whole, VZV genome sequences were highly conserved among the strains. At the level of full nucleotide sequences, SuduVax was the most similar to VarilRix, followed by vOka, VariVax and pOka (Table 2). Similar results were obtained when the genetic distances were calculated using concatenated non-coding nucleotide sequences or amino acid sequences. The average distance between SuduVax and three vaccine strains at the full nucleotide level was calculated to be 0.20 ± 0.05 × 10^-3^, which was < 10% of the average distance between SuduVax and 20 clinical strains (2.08 ± 0.39 × 10^-3^, Table 2). Among the clinical strains except for pOka, strain 8 was the most similar to SuduVax.

**Table 2:** Genetic distances between SuduVax and other VZV strains

	Nucleotide (×10^-3^)		Amino acid (×10^-3^)
		
Strains	Full	Noncoding	
Dumas	2.31	3.79	3.09
M2DR	1.87	2.77	2.57
CA123	2.34	3.50	2.75
SD	2.24	2.84	3.04
Kel	2.26	2.70	3.27
11	2.10	3.87	3.09
22	2.12	4.08	2.96
03-500	2.30	3.80	2.80
36	2.18	2.63	3.06
49	2.18	2.70	4.49
8	1.83	2.19	2.35
32p5	2.14	2.77	3.09
32p22	2.19	2.77	4.27
32p72	2.26	2.70	5.03
NH29_3	2.11	3.13	2.85
SVETA	2.15	3.43	3.01
MSP	2.21	3.35	2.93
BC	2.11	2.62	3.09
HJ0	2.20	4.00	2.98
pOka	0.53	0.58	0.97
vOka	0.19	0.15	0.57
VarilRix	0.16	0.07	0.47
VariVax	0.25	0.44	0.63

Average			
Vaccine	0.20	0.22	0.56
Clinical	2.08	3.01	3.08

### Mutations found in SuduVax ORFs

**Figure 3:**
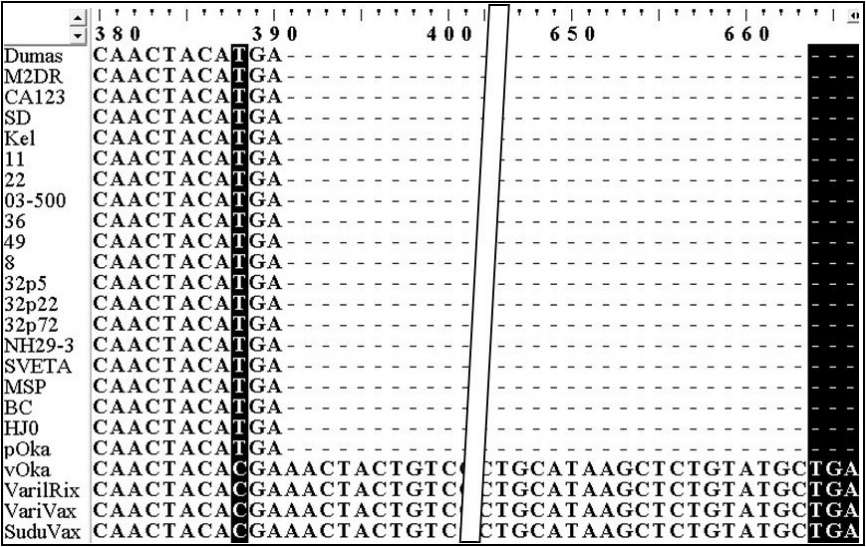
**Read-through mutation in ORF0 of SuduVax and Oka vaccine strains**. ORF0 sequences of 24 VZV strains were extracted and aligned using the ClustalW program. Substitution of T388C and putative downstream new stop codon TGA are shaded.

SuduVax ORF0 exists as longer form due to a read-through mutation. The stop codon TGA (nucleotide position 388-390) was mutated to CGA coding for Arg. A putative stop codon TGA was found downstream and overlapped with ORF1 (Figure 3). This extended ORF0 encoded a new protein with 221 amino acid residues. The same read-through mutation was found in other vaccine strains, vOka, VarilRix and VariVax. All clinical strains including pOka contained 390 bp-long ORF0 coding for 129 amino acids.

Compared to the reference strain Dumas, the lengths of ORF17 and ORF56 of the strain SuduVax were 3 bp short due to deletion of TCA at position 367 to 369 and TCT at position 658 to 660, respectively. Both deletions resulted in deletion of amino acid S residue. On the other hand, insertion of three nucleotides ATG at position 27 was found in ORF60 of the strain SuduVax. Interestingly, the aforementioned two deletions and one insertion were also found in all Oka strains including pOka. SuduVax as well as Oka strains were found to have a15 bp (AACATTTCAGGGTCA) shorter ORF29 than most clinical isolates that contain two tandem reiterations of this 15 bp sequence. Among the clinical strains, M2DR, CA123 and 8 contained only one copy of the 15 bp element in ORF29. Strains M2DR and 8 shared the same length for ORF60 with Oka and SuduVax strains. Table 3 summarizes the insertion and deletion mutations found in SuduVax.

**Table 3:** Deletions and insertion found in SuduVax

Mutation	Nucleotide	Amino acid	ORF	Also found in
Deletion	TCA	S	ORF17	4Oka^1^

	AACATTTCAGGGTCA	NISGS	ORF29	4Oka, M2DR, CA123, 8

	TCT	S	ORF56	4Oka

Insertion	ATG	M	ORF60	4Oka, M2DR, 8

^1^pOka, vOka, VariVax, and VarilRix

## Discussion

VZV strain SuduVax has been used by a Korean pharmaceutical company to produce live attenuated vaccine for chickenpox since 1994. Although its efficacy and safety have been proven in the marketplace, molecular characteristics of the vaccine strain have not been available. In this study sequencing and analyses of the nucleotide sequence of the Korean varicella vaccine strain SuduVax were undertaken.

In the original paper on the first complete sequencing of VZV strain Dumas [2], 71 ORFs were proposed. However, the information obtained from the NCBI GenBank database for Dumas (NC_001348) identifies 73 ORF if three ORFs located in TRS are counted as separate ORFs. Sequencing of two Oka-derived vaccine strains, VarilRix (DQ008354) and VariVax (DQ008355), identified 72 ORFs [5]. A Blast search using these three strains as queries produced 74 possible ORFs for VZV. We were presently able to locate ORF45 (position 81,523- 82,593) to Dumas and ORF33.5 to VarilRix (position 60,257 - 61,165) and VariVax (60,254 - 61,162). Extended from of ORF0 due to read-through mutation was identified in SuduVax as well as in Oka vaccine strains (see below). Using these reference strains Dumas and VarilRix as queries, we were able to identify and locate 74 ORFs in the genome of the strain SuduVax as well as in other 23 VZV strains analyzed in this study.

Phylogenetic analysis using the full nucleotide sequences of 24 VZV strains identified five distinct clades, consistent with previous findings [9,10]. Phylogenetic trees constructed with concatenated amino acid sequences and coding nucleotide sequences also revealed five clades with the same members. The tree built using non-coding nucleotide sequences appeared similar to the other trees, except that the strains 8 and M2DR did not form a clear clade as in other trees. SuduVax co-clustered with Oka strains and this clade consisted exclusively of isolates from Japan and Korea in clade 2. SuduVax shares the minimum complement of single nucleotide polymorphism at 27 positions [10] with other members of the clade 2. Various genotyping methods using limited genetic information of VZV strains have been proved to represent genotyping using full genome information [11-15]. Any genotyping method unequivocally placed SuduVax to the same genogroup with Oka strains as in phylogenetic trees based on full or near-full genetic information (data not shown).

It is not presently certain, because of the lack of full genome sequences from other Asian isolates, whether this clade 2 could be extended to include isolates from other Asian countries or whether it is confined to isolates from Japan and Korea only. However, available data based on partial nucleotide sequences or restriction fragment length polymorphism suggest that all Korean isolates and Chinese isolates form a clade with Japanese isolates [16,17]. Thus, it is possible that the clade 2 could be extended to include China, which is geographically close to Japan and Korea.

Coding sequences occupy approximately 91% of the VZV genome and reflect most of the sequence information of the whole genome. Thus, it was expected that the phylogenetic trees based on the coding sequences are very similar to the trees based on the full nucleotide sequences. We found that the coding sequence trees and amino acid trees were similar to the full nucleotide trees. Noncoding sequences were found to be interspersed between coding sequences or ORFs, accounting for approximately 9% of the VZV genome. The phylogenetic trees based on VZV noncoding sequences are not different from those based on full or coding nucleotide sequences or amino acid sequences. One notable difference is the location of pOka within clade 2. In full or coding sequence trees, pOka was separated from four vaccine strains to form two independent subclades within clade 2. On the contrary, pOka did not form a subclade separated from vaccine strains in noncoding sequence trees. pOka is a clinical strain. Thus, coding sequences or amino acid sequences of VZV genome may provide information distinguishing vaccine strains from clinical strains, while noncoding sequences does not.

Phylogenetic analyses using the nucleotide sequences of individual ORFs suggested 12 ORFs may be important in distinguishing vaccine strains from clinical strains. Yamanish identified 23 ORFs that are different between pOka and Oka vaccine [6], including 12 ORFs identified in this study. Moreover, our preliminary studies of single nucleotide polymorphism among the full genomic DNA sequences of the 24 VZV strains revealed 12 ORFs that may be characteristic for vaccine strains and these 12 ORFs coincide with the above-mentioned 12 ORFs [manuscript in preparation].

ORF0, also known as ORFS/L, is thought to be essential for VZV growth and encodes a membrane protein with 129 amino acid residues, which is possibly involved in vesicular trafficking and altering cell adhesion molecules in infected cells [18,19]. ORF0 in SuduVax was determined to possess an extended C-terminal sequence due to a read-through mutation of its original stop codon TGA to CGA coding for Arg. The nearest downstream stop codon TGA was found to overlap with ORF1 and the extended ORF0 is expected to code for a new protein with 221 amino acid residues. Interestingly, this read-through mutation was also found in the three Oka-derived vaccine strains, while the stop codons were found to be unaltered in all of the clinical strains including the parent Oka strain. In cells infected with vOka, the extended form of ORF0 protein with 221 amino acid residues and its spliced form with 155 amino acid residues are expressed [20]. Since other vaccine strains, including SuduVax, share 100% identical nucleotide sequences within and downstream of ORF0 up to the new stop codon, both forms of the extended ORF0 proteins are expected to be expressed in permissive cells infected with SuduVax. Thus, read-through mutation in ORF0 might be an important feature distinguishing vaccine strains from clinical strains.

Besides the read-through mutation in ORF0, SuduVax share same mutational events in ORFs 17, 29, 56 and 60 with Oka strains. ORF17 codes for an mRNA-specific RNase [21] and ORF29 encodes single strand DNA binding protein via its zinc-finger domain [22]. The function of ORF56 has not been well characterized, but its gene product is reported to co-localize with regulatory protein ICP22 and nuclear protein UL3 in small, dense nuclear bodies (NCBI, http://www.ncbi.nlm.nih.gov/pubmed?Db=gene&Cmd=retrieve&dopt=full_report&list_uids=1487683. The gene product of ORF60 is glycoprotein L, which acts as a chaperon for glycoprotein H [23]. Three bp deletions were found in ORFs 17 and 56, and an insertion of 3-bp was found in ORF60. While most of the clinical strains contain two tandem copies of 15 bp (AACATTTCAGGGTCA) elements in ORF29, while the SuduVax and Oka strains contain only one copy of this 15 bp element. Of these four deletion and insertion events, two events (ORFs 29, 60) are shared with the clinical strains 8 and M2DR, and one event (ORF29) is also found in the strain CA123. Since these deletion and insertion events are also found in some of the clinical strains including pOka, they by themselves may not be important in attenuation, although it is still possible that they, in combination with other events such as read-through mutation in ORF0, may play some roles in attenuation of vaccine strains.

## Conclusion

We obtained and analyzed full nucleotide sequence of the Korean vaccine strain SuduVax. SuduVax was shown to be genetically most similar to Oka-derived vaccine strains. We are now comparing the SuduVax nucleotide and amino acid sequences with those of other vaccine and clinical strains. Further comparative genomic and bioinformatics analyses will help to elucidate the molecular basis of the attenuation of the VZV vaccine strains.

## Materials and methods

### Virus and DNA sequencing

DNA of the VZV strain SuduVax was extracted from commercial vials of SuduVax™ with QIAamp DNA Mini Kit (QIAGEN) at a concentration of 5.5 μg/100 μL. The DNA sequence was determined by the high throughput sequencing method using a Genome Sequencer FLX Titanium System of Roche Diagnostics, serviced by Macrogen. Sequence fragments (n = 23,722) with an average length of approximately 400 bp were obtained and these were assembled and viewed using the Consed program http://bozeman.mbt.washington.edu/consed/consed.html. The average quality of the sequence fragments was more than 99.99%. A total of 99.38% of the 124,759 sequences aligned with the derived consensus sequence and the average coverage was 83 reads per nucleotide. These were aligned against reference strain Dumas (NC_001348) and vaccine strain VarilRix (DQ008354). The gaps between the contigs were filled by polymerase chain reaction sequencing using primers whose sequences were obtained from the adjacent contigs. The completed sequence was deposited into NCBI GenBank (accession number JF306641).

### Allocation of ORFs

ORFs of the strain SuduVax in the full genome sequence was located by Blast search against two reference strains Dumas (NC_001348) and VarilRix (DQ008354). Complementary determining sequences (CDSs) of the reference strains were extracted using FeatureExtract 1.2 Server program http://www.cbs.dtu.dk/services/FeatureExtract/ and used as query. The resulting data included the first and last nucleotide positions of the each ORF in the strain SuduVax genome and direction of the ORFs. The ORF information was verified by ORF finding programs such as CLC Sequence Viewer (version 6.4, http://www.clcbio.com/index.php) and ORF Finder provided by NCBI. When the results of Blast search did not coincide with those of ORF finding programs, the nucleotide sequences of the corresponding ORFs were examined with BioEdit Sequence Alignment Editor (Department of Microbiology, North Carolina State University, version 7.0.5.3, http://www.mbio.ncsu.edu/BioEdit/bioedit.html) and manually edited to locate the position of the start and stop codons. Finally all the allocated ORFs were confirmed by identification of the translated amino acid sequences.

### Phylogenetic analysis

Nucleotide sequences of the VZV full genome other than SuduVax were obtained directly from GenBank database (Table 1). For each VZV strain, all ORF sequences were cut and pasted to generate a concatenated coding sequence. Similarly, all inter-ORF sequences were cut and pasted to build a concatenated noncoding sequence. Amino acid sequences were obtained by translation of the corresponding ORFs and pasted to generate a concatenated sequence harbouring all 74 ORFs. These full or concatenated nucleotide or concatenated amino acid sequence of the 24 VZV strains were multiple-aligned using the ClustalW program (ver 2.0.1) followed by manual editing. The resulting out-files were used to calculate genetic distances using Dnadist (for nucleotide) or Protdist (for amino acid) program included in Phylip package (version 3.69, http://evolution.genetics.washington.edu/phylip.html). Distance matrix was obtained by Kimura-2-parameter for nucleotide or Jones-Taylor-Thornton method for amino acid. Cluster analysis was performed by neighbour-joining (NJ) and maximum-likelihood (ML) method and resulting tree files were viewed by Treeview program (version 1.6.6). The significance of the phylogenetic trees was verified by bootstrap analysis. Phylogenetic trees were constructed from 1,000 replicates generated by the Seqboot program and the consensus tree was identified by the Consense program.

## Abbreviations

HEL: Human embryonic lung; ML: Maximum likelihood; NJ: Neighbour-joining; ORF: Open reading frame; VZV: Varicella-zoster virus.

## Competing interests

The authors declare that they have no competing interests.

## Authors' contributions

HSP, SYP, KML and CHL conceived and designed the experiments. JIK, GSJ, YYK, GYJ, HSK, WDW and CHL performed experiments and analyzed the data. SYP, GHK, and SNK prepared samples. JIK, GSJ and CHL wrote the paper. All authors read and approved the final manuscript.

## References

[B1] Weller TH Serial propagation in vitro of agents producing inclusion bodies derived from varicella and herpes zoster Proc Soc Exp Biol Med 1953 83 2 340 346 13064265 10.3181/00379727-83-20354

[B2] Davison AJ Scott JE The complete DNA sequence of varicella-zoster virus J Gen Virol 1986 67 Pt 9 1759 1816 3018124 10.1099/0022-1317-67-9-1759

[B3] Argaw T Cohen JI Klutch M Lekstrom K Yoshikawa T Asano Y Krause PR Nucleotide sequences that distinguish Oka vaccine from parental Oka and other varicella-zoster virus isolates J Infect Dis 2000 181 3 1153 1157 10.1086/315335 10720545

[B4] Gomi Y Sunamachi H Mori Y Nagaike K Takahashi M Yamanishi K Comparison of the complete DNA sequences of the Oka varicella vaccine and its parental virus J Virol 2002 76 22 11447 11459 10.1128/JVI.76.22.11447-11459.2002 12388706 PMC136748

[B5] Tillieux SL Halsey WS Thomas ES Voycik JJ Sathe GM Vassilev V Complete DNA sequences of two oka strain varicella-zoster virus genomes J Virol 2008 82 22 11023 11044 10.1128/JVI.00777-08 18787000 PMC2573284

[B6] Yamanishi K Molecular analysis of the Oka vaccine strain of varicella-zoster virus J Infect Dis 2008 197 Suppl 2 S45 48 18419407 10.1086/522122

[B7] Hwang KK Park SY Kim SJ Ryu YW Kim KH Restriction fragment length polymorphism analysis of Varicella-Zoster Virus isolated in Korea J Kor Soc Virol 1991 21 2 201 210

[B8] Sohn YM Park CY Hwang KK Woo GJ Park SY Safety and immunogenicity of live attenuated Varicella Virus Vaccine (MAV/06) J Kor Pediatr Soc 1994 37 1405 1413

[B9] Loparev V Martro E Rubtcova E Rodrigo C Piette JC Caumes E Vernant JP Schmid DS Fillet AM Toward universal varicella-zoster virus (VZV) genotyping: diversity of VZV strains from France and Spain J Clin Microbiol 2007 45 2 559 563 10.1128/JCM.01738-06 17135433 PMC1829061

[B10] Breuer J Grose C Norberg P Tipples G Schmid DS A proposal for a common nomenclature for viral clades that form the species varicella-zoster virus: summary of VZV Nomenclature Meeting 2008, Barts and the London School of Medicine and Dentistry, 24-25 July 2008 J Gen Virol 2010 91 Pt 4 821 828 20071486 10.1099/vir.0.017814-0 PMC2888159

[B11] Barrett-Muir W Scott FT Aaby P John J Matondo P Chaudhry QL Siqueira M Poulsen A Yaminishi K Breuer J Genetic variation of varicella-zoster virus: evidence for geographical separation of strains J Med Virol 2003 70 Suppl 1 S42 S47 12627486 10.1002/jmv.10319

[B12] Faga B Maury W Bruckner DA Grose C Identification and mapping of single nucleotide polymorphisms in the varicella-zoster virus genome Virology 2001 280 1 1 6 10.1006/viro.2000.0775 11162813

[B13] Loparev VN Gonzalez A Deleon-Carnes M Tipples G Fickenscher H Torfason EG Schmid DS Global identification of three major genotypes of varicella-zoster virus: longitudinal clustering and strategies for genotyping J Virol 2004 78 15 8349 8358 10.1128/JVI.78.15.8349-8358.2004 15254207 PMC446121

[B14] Peters GA Tyler SD Grose C Severini A Gray MJ Upton C Tipples GA A full-genome phylogenetic analysis of varicella-zoster virus reveals a novel origin of replication-based genotyping scheme and evidence of recombination between major circulating clades J Virol 2006 80 19 9850 9860 10.1128/JVI.00715-06 16973589 PMC1617253

[B15] Wagenaar TR Chow VT Buranathai C Thawatsupha P Grose C The out of Africa model of varicella-zoster virus evolution: single nucleotide polymorphisms and private alleles distinguish Asian clades from European/North American clades Vaccine 2003 21 11-12 1072 1081 10.1016/S0264-410X(02)00559-5 12559782

[B16] Liu J Wang M Gan L Yang S Chen J Genotyping of clinical varicella-zoster virus isolates collected in China J Clin Microbiol 2009 47 5 1418 1423 10.1128/JCM.01806-08 19244468 PMC2681844

[B17] Kim KH Choi YJ Song KH Park WB Jeon JH Park SW Kim HB Kim NJ Oh MD Genotype of varicella-zoster virus isolates in South Korea J Clin Microbiol 2011 49 5 1913 1916 10.1128/JCM.02356-10 21411584 PMC3122681

[B18] Kemble GW Annunziato P Lungu O Winter RE Cha TA Silverstein SJ Spaete RR Open reading frame S/L of varicella-zoster virus encodes a cytoplasmic protein expressed in infected cells J Virol 2000 74 23 11311 11321 10.1128/JVI.74.23.11311-11321.2000 11070031 PMC113236

[B19] Zhang Z Rowe J Wang W Sommer M Arvin A Moffat J Zhu H Genetic analysis of varicella-zoster virus ORF0 to ORF4 by use of a novel luciferase bacterial artificial chromosome system J Virol 2007 81 17 9024 9033 10.1128/JVI.02666-06 17581997 PMC1951468

[B20] Koshizuka T Ota M Yamanishi K Mori Y Characterization of varicella-zoster virus-encoded ORF0 gene-comparison of parental and vaccine strains Virology 2010 405 2 280 288 10.1016/j.virol.2010.06.016 20598727

[B21] Sato H Callanan LD Pesnicak L Krogmann T Cohen JI Varicella-zoster virus (VZV) ORF17 protein induces RNA cleavage and is critical for replication of VZV at 37 degrees C but not 33 degrees C J Virol 2002 76 21 11012 11023 10.1128/JVI.76.21.11012-11023.2002 12368344 PMC136605

[B22] Stallings CL Duigou GJ Gershon AA Gershon MD Silverstein SJ The cellular localization pattern of Varicella-Zoster virus ORF29p is influenced by proteasome-mediated degradation J Virol 2006 80 3 1497 1512 10.1128/JVI.80.3.1497-1512.2006 16415026 PMC1346923

[B23] Duus KM Hatfield C Grose C Cell surface expression and fusion by the varicella-zoster virus gH:gL glycoprotein complex: analysis by laser scanning confocal microscopy Virology 1995 210 2 429 440 10.1006/viro.1995.1359 7618278

